# The effect of drug ionization on lipid-based formulations for the oral delivery of anti-psychotics

**DOI:** 10.5599/admet.830

**Published:** 2020-07-17

**Authors:** Tahlia R Meola, Kara Paxton, Paul Joyce, Hayley B Schultz, Clive A Prestidge

**Affiliations:** 1 UniSA: Clinical and Health Sciences, University of South Australia, City West Campus, Adelaide, South Australia 5000, Australia; 2 ARC Centre of Excellence in Convergent Bio-Nano Science & Technology, University of South Australia, City West Campus, Adelaide, South Australia 5000, Australia

**Keywords:** silica, nanoparticles, lurasidone, risperidone, pKa, precipitation, interactions, dissolution, lipolysis

## Abstract

Lipid-based formulations (LBFs) are well-known to improve the oral bioavailability of poorly water-soluble drugs (PWSDs) by presenting the drug to the gastrointestinal environment in a molecularly dispersed state, thus avoiding the rate-limiting dissolution step. Risperidone and lurasidone are antipsychotics drugs which experience erratic and variable absorption, leading to a low oral bioavailability. The aim of this research was to develop and investigate the performance of risperidone and lurasidone when formulated as an emulsion and silica-lipid hybrid (SLH). Lurasidone and risperidone were dissolved in Capmul® MCM at 100% and 80% their equilibrium solubility, respectively, prior to forming a sub-micron emulsion. SLH microparticles were fabricated by spray-drying a silica stabilised sub-micron emulsion to form a solid powder. The performances of the formulations were evaluated in simulated intestinal media under digesting conditions, where the emulsion and SLH provided a 17-fold and 23-fold increase in LUR solubilisation, respectively. However, the performance of RIS was reduced by 2.2-fold when encapsulated within SLH compared to pure drug. Owing to its pKa, RIS adsorbed to the silica and thus, dissolution was significantly hindered. The results reveal that LBFs may not overcome the challenges of all PWSDs and physiochemical properties must be carefully considered when predicting drug performance.

## Introduction

Adherence to antipsychotic medication is a significant challenge for patients, whereby research suggests at least half of patients are non-compliant, leading to illness relapse, hospital remittance or suicide [1]. Due to the chronic nature of schizophrenia, the oral route of administration is preferred. However, as a class, antipsychotic drugs are generally poorly water-soluble, resulting in limited absorption and sub-optimal oral bioavailability when administered as a conventional tablet [2, 3].

The ability of lipids to improve the oral absorption and bioavailability of poorly water-soluble drugs is well-established in literature [4, 5]. The enhanced performance provided by lipid-based formulations (LBFs) can be attributed to the following proposed mechanisms: (i) lipids in the gastrointestinal tract hinder gastric emptying from the stomach, therefore increase transit time of the drug in the small intestine, allowing greater absorption to occur [6, 7]; (ii) drug can be encapsulated and pre-solubilised in lipid and presented to the gastrointestinal tract in its molecularly dispersed state, therefore drug absorption is no longer limited by its dissolution rate [8, 9], and (iii) exogenous lipids stimulate the release of bile and pancreatic lipase from the gall bladder and pancreas, respectively, which triggers the production of solubilising species that can further solubilise the drug and be transported across the epithelium via enterocytes [6, 7]. Despite the magnitude of advantages, LBFs account for only 2-4% of commercialised pharmaceutical formulations [10, 11]. This is likely attributed to disadvantages associated with their liquid-state, such as poor physical stability, drug leakage, oxidation of lipid excipients and drug precipitation [12, 13].

Silica-lipid hybrid (SLH) microparticles are a solid-state formulation which encapsulates liquid-lipid within the porous matrix. SLH were developed to overcome liquid-state limitations by combining the solubilising effects of lipids with the stabilising effects of silica nanoparticles, to provide enhanced physical stability and superior performance [14]. This LBF has proven successful for numerous poorly water-soluble compounds such as ibuprofen, celecoxib, abiraterone acetate and simvastatin [15-18]. SLH microparticles are fabricated by spray-drying a silica nanoparticle-stabilised Pickering emulsion to form a solid nanostructured powder. Owing to a highly porous matrix, lipase enzymes can readily digest the lipid, thus promoting drug release and absorption [19].

Lurasidone (LUR) and risperidone (RIS) are two orally administered poorly water-soluble antipsychotic drugs which are stipulated to benefit from reformulation within LBFs. LUR and RIS are weak monoprotic and diprotic basic compounds, respectively, with pH dependent solubility. The p*K*a of LUR is 7.6, whereas RIS has two p*K*a values of 3.1 and 8.2 [20, 21]. Therefore, at neutral pH, simulating the environment within the small intestine, LUR is approximately 40% positively ionised, whereas RIS is approximately 80% positively ionised. Subsequently, only 9-19% of the administrative oral LUR dose (ranging between  20-160 mg daily) is absorbed, when co-administered with a meal [22, 23]. In comparison, a daily oral RIS dose of 2-16 mg has an oral bioavailability of approximately 70%, with erratic and variable absorption of up to 25% between patients [24]. Thus, the reliance of patient compliance for co-administering medication with food, along with the unpredictable bioavailability, is highly problematic when dosing anti-psychotics, which highlights the critical need for reformulation of LUR and RIS in delivery systems that enhance absorption while reducing variability.

In this study, LUR and RIS will be hosted within the lipid component of SLH microparticles in an attempt to improve their solubilisation in simulated intestinal conditions. The solubilising effects of SLH formulations will be compared to a conventional drug in lipid emulsion, and unformulated pure drug. In doing so, the impact of (i) drug ionisation and (ii) formulation physicochemical properties, on *in vitro* dissolution and solubilisation under non-digesting and digesting conditions will be critically evaluated. It was hypothesised that fabrication of RIS and LUR within LBFs, specifically SLH microparticles, will maximise drug solubilisation, under simulated intestinal conditions in the fasted state, due to the ability for lipid species to maintain the drug in a molecular form. By investigating the impact of drug ionisation on solubilisation behaviour, new insights will be derived regarding the key physicochemical properties of solid-state LBFs required for improving oral delivery performance.

## Experimental

### Materials

Risperidone (RIS), lurasidone (LUR) and ziprasidone powders were purchased from Hangzhou Dayangchem Co. Ltd (Hangzhou, China). Capmul® MCM (glyceryl mono- and dicaprylate) was a gift from Abitec Corporation (Wisconsin, USA). Hydrophilic fumed silica nanoparticles (Aerosil® 300) were supplied by Evonik Degussa (Essen, Germany). Soybean lecithin was purchased from BDH Merck (Sydney, Australia). Sodium dihydrogen phosphate (NaH_2_PO_4_), orthophosphoric acid, potassium dihydrogen phosphate (KH_2_PO_4_), sodium hydroxide pellets (NaOH), Tween 80, and Trizma® maleate, sodium chloride (NaCl), calcium chloride dehydrate (CaCl_2_.2H_2_O), egg lecithin (consisting of 60% phosphatidylcholine from dried egg yolk), sodium taurodeoxycholate (NaTDC), 4-bromophenylboronic acid (4-BBA) and myristic acid were purchased from Sigma Aldrich (Sydney, Australia) and porcine pancreatin extracts from MP Biomedicals (Sydney, Australia). High purity (Milli-Q) water and HPLC grade solvents were used during the study.

### High-performance liquid chromatography (HPLC)

The concentrations of RIS and LUR were quantified using high performance liquid chromatography (HPLC) analysis on a Shimadzu Prominence Ultra-Fast Liquid Chromatograph (UFLC XR) system, equipped with a Phenomenex Luna 5 μm C18 100 Å (250 mm × 4.6 mm) column, maintained at 40 °C. An isocratic elution method was employed at a flow rate of 1 mL/min, using a mobile phase comprising of 0.05 M NaH_2_PO_4_ buffer (adjusted to pH 3 using orthophosphoric acid) and methanol (40:60 and 25:75 v/v for RIS and LUR, respectively). RIS was detected at a wavelength of 280 nm at a retention time of 3.8 min, whereas LUR was detected at 231 nm with an average retention time of 5.8 min. Linear calibration curves were generated for both compounds using chromatographic peak area against standard concentrations over a range of 0.02-20 μg/mL. Quality control standards were analysed with every sample batch. The precision and accuracy of inter- and intra-day analyses were within an acceptable range of less than 10%.

### Liquid chromatography-mass spectrometry (LCMS)

The concentration of LUR for drug solubilisation studies was determined using liquid chromatography-mass spectrometry (LCMS) on a Shimadzu 8030 mass spectrometer (Kyoto, Japan). A 10 μL sample was injected into a Phenomenex Kinetex 2.6 μm C18 100 Å (50 mm × 3 mm) column, maintained at 40 °C. A gradient elution method consisting of (A) Milli-Q water + 0.1% formic acid and (B) acetonitrile + 0.1% formic acid was employed at a flow rate of 0.4 mL/min. Multiple mode monitoring was used to quantify the internal standard ziprasidone precursor at 413.2 m/z and product at 194.0 m/z. LUR precursor was detected at 493.2 m/z and product at 166.1 m/z. Linear calibration curves were generated over concentrations ranging from 0.2-1 μg/mL by plotting peak area ratio of analyte: internal standard against known concentrations.

### Preparation of drug loaded lipid emulsions

The equilibrium solubilities of LUR and RIS in Miglyol® 812, Capmul® MCM and soybean oil were determined by adding excess drug to a known quantity of lipid. The drug-lipid suspensions were sonicated for 10 min to facilitate dissolution, prior to rotating for 72 h at room temperature, protected from light. Aliquots were analysed at 24, 48 and 72 h to determine whether equilibrium had been reached. Samples were centrifuged at 29066 × g for 20 min at 24 °C to separate any undissolved drug. Solvent extraction was performed to extract the drug from the lipid supernatant by diluting the lipid supernatant with methanol (100-fold dilution) and sonicating for 10 min, prior to further centrifugation at 29066 × g for 10 min at 24 °C for phase separation. The resulting supernatant was diluted appropriately and analysed via HPLC.

Submicron lipid emulsions with drug loaded into lipid at 80% and 100% the equilibrium solubility for RIS and LUR, respectively, were formulated. Soybean lecithin (6% w/w) was dissolved in Capmul® MCM with the aid of sonication. Drug was dissolved in the lipid prior to the addition of Milli-Q water. The coarse emulsions were sonicated for 1 h to form submicron emulsions.

### Fabrication of drug loaded silica-lipid hybrid microparticles

SLH microparticles were fabricated following a two-stage process modified from Tan *et al.* [14]. A silica dispersion (5% w/v) was added to the drug-lipid emulsions to achieve a lipid to silica ratio of 1:1 and 1:2 for RIS and LUR, respectively. The silica-stabilised Pickering emulsions were stirred overnight at room temperature prior to spray-drying to form solid SLH microparticles (Mini Sprayer B-290, BÜCHI Labortechnik AG, Switzerland). Processing parameters were as follows: inlet temperature of 160 °C, aspirator setting at 100%, pump setting at 20% and an outlet temperature of approximately 65 °C.

### Physicochemical characterisation

The drug content of the SLH formulations were determined by a solvent extraction method. Approximately 10 mg of powder was dispersed in 10 mL of methanol. The dispersion was sonicated for 45 min and centrifuged at 29066 × g for 20 min at 24 °C, prior to dilution for HPLC analysis.

The particle size of the SLH formulations were analysed using a Malvern Mastersizer 3000 Hydro LV (Malvern, United Kingdom). Formulations were added to the sample vessel until a laser obscuration of 10-20% was achieved. A particle refractive index of 1.54 was utilised. Results are expressed as volume diameter (μm) at 50% cumulative volume (D (v.0.5)).

The surface morphologies of the formulations were analysed through high-resolution analytical scanning electron microscopy (SEM) (Carl Ziess Microscopy Merlin with a Gemini II column, Germany) at an accelerating voltage of 8 kV and spot size of 3-10 μm. Samples were attached to double-faced adhesive tape and sputter coated with gold (5-10 nm thickness) prior to imaging.

The crystallinity of encapsulated drug was analysed via differential scanning calorimetry (DSC Q100, TA instruments, Australia). Approximately 2 mg of SLH was hermetically sealed in aluminium pans and heated at a rate of 10 °C/min from 25 °C to 170 °C for LUR and 200 °C for RIS, under nitrogen (80 mL/min).

### In vitro dissolution and release studies

*In vitro* dissolution and release studies were performed under non-sink conditions using a USP type II apparatus operating at 50 rpm. Formulations equivalent to 10 mg RIS and 5 mg LUR were added to pH 7.4 phosphate buffer containing 1% w/v Tween 80, maintained at 37 °C. Aliquots were taken at pre-determined time points and replaced with an equal volume of fresh media. Samples were immediately centrifuged at 29066 × g for 10 min at 24 °C. The supernatant was diluted appropriately with mobile phase for HPLC analysis.

To study the interaction between RIS and silica, excess RIS was added to the pH 7.4 phosphate buffer containing 1% w/v Tween 80 in a glass vial and sonicated for 1 h to facilitate dissolution. Temperature was maintained at 37 °C by manual adjustment. Vials were rotated at 37 °C for an additional 20 min and an aliquot was taken. Aerosil® 300 silica nanoparticles (equivalent mass to SLH content) were added to the vial and further aliquots were taken at pre-determined time points. Samples were centrifuged at 29066 × g for 10 min at 24 °C, and the resulting supernatant was diluted prior to HPLC analysis.

### Solubilisation studies under digesting conditions during in vitro lipolysis

Simulated fasted-state intestinal media was prepared using a method previously developed by Sek *et al.* [25]. Mixed fasted-state micelles with a bile salt: phosphatidylcholine concentration of 5 mM: 1.25 mM were prepared via the following procedure. Egg lecithin was dissolved in chloroform prior to evaporation to form a thin, dry lecithin film. NaTDC and digestion buffer (consisting of 50 mM Trizma® maleate, 150 mM sodium chloride and 5 mM calcium chloride) were added, and the mixture was left to stir overnight to produce a clear micellar solution.

*In vitro* lipolysis was performed using a 902 Titrando pH-stat titration apparatus (Metrohm, Switzerland). Formulations equivalent to 5 mg LUR or RIS (357 μL for LUR emulsion, 667 mg for LUR SLH, 160 uL for RIS emulsion and 305 mg for RIS SLH) were added to 18 mL of fasted-state micelles, stirred and maintained at 37 °C in a jacketed vessel. Lipolysis was initiated by the addition of 2 mL of pancreatic lipase (1000 TBU). Fatty acids produced during lipolysis were automatically titrated with 0.6 M NaOH to maintain a constant pH of 6.5 in the digestion medium. The cumulative volume of NaOH was converted into percentage lipolysis using equation 1, where V is volume, C is concentration, MW is molecular weight and m is the moles of fatty acids produced.


(1)

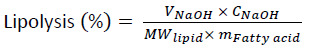



To monitor drug solubilisation, aliquots were taken at pre-determined time points up to 60 min and added to centrifuge tubes containing 0.5 M 4-BBA to inhibit lipase action. Samples were centrifuged at 35170 × g for 1 h at 37 °C to separate the solubilised aqueous phase and precipitated pellet phase. The aqueous phase was then extracted with acetonitrile, sonicated for 10 min and centrifuged again at 29066 × g for 10 min at 24 °C. Samples were diluted appropriately for analysis via HPLC and LCMS for RIS and LUR, respectively.

### Zeta potential measurements of silica: drug: fatty acid complexes

Zeta potential measurements were performed using phase analysis light scattering (PALS; Zetasizer Nano ZS, Malvern Instruments, Worcestershire, UK), to investigate possible interactions between the porous silica particles, drug molecules and fatty acids. Firstly, the zeta potential of spray dried fumed silica particles, dispersed in lipolysis media at a concentration of 50 μg/mL, was assessed. Then, drug dissolved in 50: 50 methanol: water mix was added to this dispersion at a final concentration of 5 μg/mL and the zeta potential was reanalysed. Finally, the silica: drug complex was spiked with myristic acid (medium chain, C12 fatty acid; 50 μg/mL) and the zeta potential was again reanalysed. Zeta potential measurements are presented as mean ± standard deviation of 3 replicate measurements.

### Statistical Analysis

Experimental data were statistically analysed using an unpaired student’s t-test in GraphPad. Data was considered statistically significance when *p < 0.05.*

## Results and discussion

### Fabrication and physicochemical characterisation of RIS and LUR formulations

**Figure 1.**
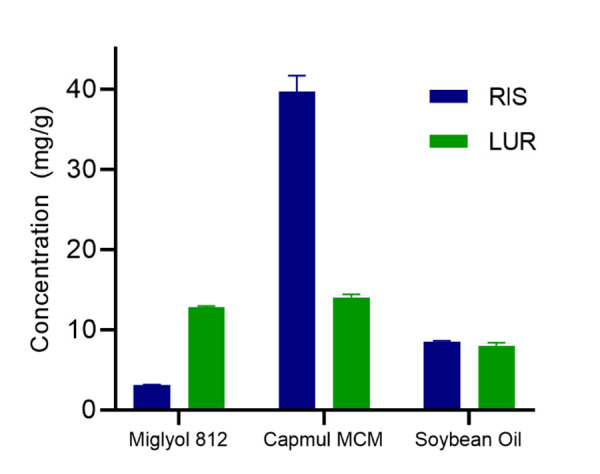
Equilibrium solubilities of RIS and LUR in various lipids. Each value represents the mean ± SD, *n* = 3.

The drug loading levels of LBFs is dependent on the solubilising capacity of the active pharmaceutical ingredient in the lipid reservoir. Therefore, the equilibrium solubilities (*S*_eq_) of LUR and RIS, at room temperature, were investigated in Miglyol® 812, Capmul® MCM and soybean oil, since each lipid comprises of varying chain lengths and compositions (Figure 1). Capmul® MCM, a mixture of medium chain mono- and di-glyceride medium chain fatty acids, provided the highest solubilisation for both compounds, reaching a solubilisation capacity of 39.7 ± 2.8 mg/g and 14.0 ± 0.6 mg/g for RIS and LUR, respectively. Miglyol® 812, a mixture of medium chain triglycerides, revealed a higher solubilisation for LUR, reaching a *S*_eq_ of 12.8 ± 0.3 mg/g, compared to 3.13 ± 0.1 mg/g for RIS. No significant difference was found between RIS and LUR in soybean oil, which is a mixture of long chain triglycerides (8.53 ± 0.2 mg/g for RIS and 7.99 ± 0.6 mg/g for LUR). Therefore, Capmul® MCM was chosen for further formulation development since it provided the highest solubilisation capacity for both RIS and LUR and allows for the highest drug loading levels within each LBF.

The clinical oral daily dose of LUR ranges from 20 – 160 mg/day, compared to 2 – 16 mg/day for RIS [23, 24]. Consequently, due to different dosing requirements, submicron emulsions were fabricated by dissolving LUR and RIS in Capmul® MCM at 100% and 80% their *S*_eq_, respectively. Table 1 summarises the drug loading levels and loading efficiencies of the investigated formulations. The submicron emulsions for LUR and RIS will be referred to as LUR emulsion and RIS emulsion, respectively, herein. The drug loading level of the RIS emulsion was 3.12 ± 0.0% w/w, compared to 1.4 ± 0.0% w/w for the LUR emulsion.

**Figure 2.**
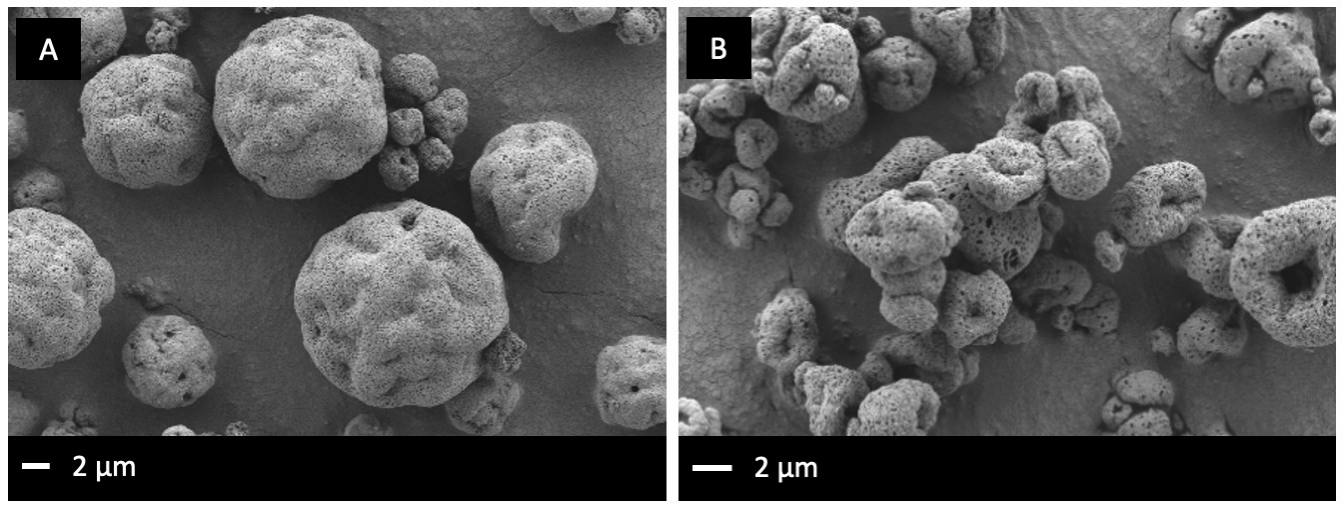
SEM images depicting the surface morphologies of **(A)** RIS-SLH, and **(B)** LUR-SLH.

SLH formulations were manufactured by spray-drying the submicron emulsion with a silica nanoparticle dispersion to form solid SLH microparticles. RIS-SLH particles comprised of a 1: 1 lipid: silica ratio and obtained a final drug load of 1.64 ± 0.2% w/w. In attempt to increase the formulation drug load of LUR-SLH to meet the higher dosing requirements, a 2:1 ratio of lipid:silica was utilised, achieving a final drug loading level of 0.75% w/w. Both SLH formulations formed free-flowing powders following the spray drying procedure. Particle size analysis after a 5 min dispersion period in aqueous media revealed the particle size of RIS-SLH to be slightly larger than LUR-SLH (D[v,0.5] of 9.39 ± 1.3 μm and 7.46 ± 1.5 μm for RIS-SLH and LUR-SLH, respectively). As demonstrated by the SEM images in Figure 2, RIS-SLH consisted of spherical porous microparticles, compared to LUR-SLH which appeared to have a collapsed porous structure. As the spray-drying parameters were consistent for both formulations, the different shape may be attributed to the different lipid:silica composition, i.e. a greater amount of lipid resulted in the collapse of the microparticles.

**Figure 3.**
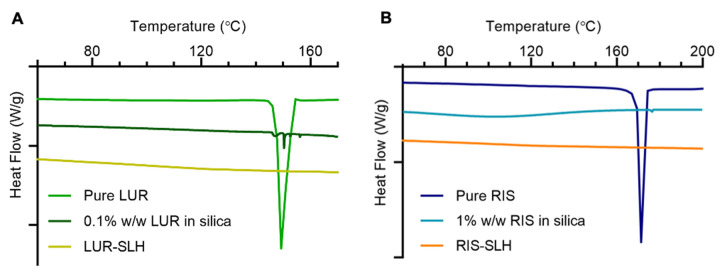
DSC thermograms of **(A)** LUR formulations and **(B)** RIS formulations.

Given LBFs are known for their ability to maintain poorly water-soluble drugs in a non-crystalline state, the degree of crystallinity of LUR and RIS encapsulated in the SLH formulations was investigated using DSC (Figure 3). The thermograms of pure RIS and LUR powders displayed strong endothermic peaks at approximately 173 °C and 150 °C, respectively, corresponding to the drug’s melting points and thus, indicating crystallinity. Due to the low drug loading levels for SLH formulations, physical mixtures containing 0.1% w/w LUR in silica and 1% w/w RIS in silica were analysed to determine whether DSC provided adequate sensitivity to detect the drug within the formulations. The characteristic peaks were present for the physical mixtures; however, the RIS endothermic peak in the physical mixture shifted to approximately 177 °C due to potential molecular interaction with the silica. The characteristic peaks were absent for RIS-SLH and LUR-SLH, confirming that the compounds were encapsulated within the silica-lipid matrix in a non-crystalline state. Although DSC was not performed on the pre-cursor submicron emulsions, it is hypothesised that both RIS and LUR within the emulsions would be non-crystalline due to being loaded at or below their equilibrium solubilities in the lipid.

### In vitro dissolution and release studies

#### Impact of LUR reformulation on dissolution and release

**Figure 4.**
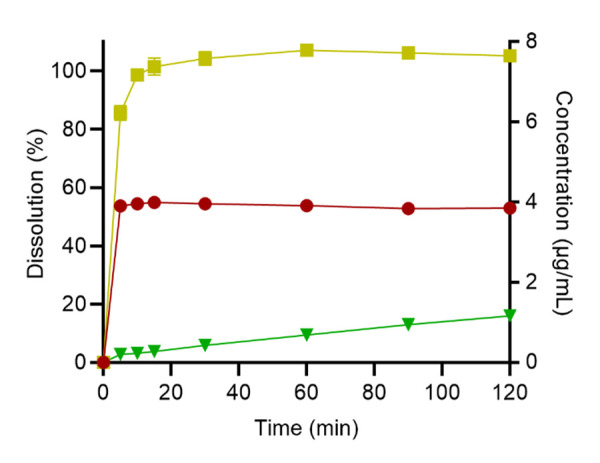
*In vitro* dissolution profiles of pure LUR [▼], LUR emulsion [●], and LUR-SLH [■]. Each value represents the mean ± SD, *n* = 3.

*In vitro* dissolution and release studies were conducted in simulated intestinal media under non-sink conditions. Pure LUR exhibited the lowest rate and extent of dissolution reaching a maximum of 16.0 ± 0.5% (1.2 μg/mL) after 2 h, reflecting the drug’s poor aqueous solubility and crystalline nature (Figure 4). When formulated as a submicron emulsion, the release of LUR was enhanced 3.4-fold, compared to pure LUR (52.9 ± 0.8% release, 3.9 μg/mL). It is postulated that this is due to the pre-solubilisation of the drug in the lipid, therefore avoiding the critical rate-limiting dissolution step. LUR-SLH displayed a rapid release of LUR, achieving complete drug release after a 10 min period. Similar to the submicron emulsion, the rapid release rate of LUR-SLH can be explained by the encapsulation of LUR in the non-crystalline state. However, it is apparent that the 1.9-fold enhancement in drug release, compared to the submicron emulsion, is due to the presence of hydrophilic silica and porous structure, aiding in drug wettability and dissolution [16].

#### Impact of RIS reformulation on dissolution and release

**Figure 5.**
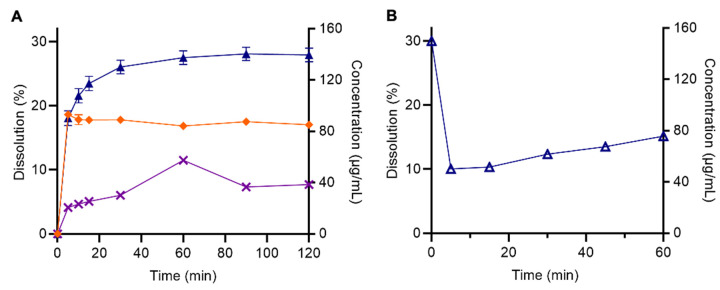
*In vitro* dissolution profiles of **(A)** pure RIS [▲], RIS emulsion [◆], and RIS-SLH [✕], and **(B)** pure RIS solution followed by the addition of silica at *t* = 0 min. Each value represents the mean ± SD, *n* = 3.

The opposite trend in performance was evident for RIS formulations, whereby the dissolution of pure RIS was superior to both LBFs, reaching 28.0 ± 1.0% (156.1 μg/mL) after a 2 h period (Figure 5). During the initial 5 min, the rate of RIS release from the RIS emulsion mimicked the release of pure RIS, prior to plateauing at 17.0 ± 0.5% (83.7 μg/mL) for the remaining experimental period. RIS-SLH exhibited the lowest extent of drug release reaching 7.7 ± 0.0% (38.5 μg/mL) after 120 min, a 4-fold reduction compared to pure drug dissolution. To investigate the reason for this poor performance, a study was conducted to determine whether any interaction between silica and RIS was present, by introducing Aerosil® silica to dissolution media containing dissolved RIS (Figure 5B). Results revealed that the addition of silica triggered a significant reduction in the concentration of RIS in solution from 28.0% to 15.2 ± 0.5%. This was hypothesised to be due to the ionic interactions and adsorption of the positively charged RIS to the negatively charged silica. Nevertheless, drug release from the RIS emulsion was inferior to pure RIS, even though silica was absent. Therefore, it is reasonable to suggest that due to the lipophilic nature of RIS (log *P* = 3.04) [21], RIS favours Capmul® MCM, resulting in less drug partitioning into the aqueous phase and reducing the overall extent of drug release. It is recognised that a limitation exists when analysing LBFs during *in vitro* release studies as no enzyme is present to digest the lipid to release the drug and thus, the formulation performance may be underestimated. Hence, *in vitro* solubilisation studies under digesting conditions were subsequently performed.

### In Vitro Solubilisation Studies under Digesting Conditions

#### Impact of LUR reformulation on lipolysis and drug solubilisation

**Figure 6.**
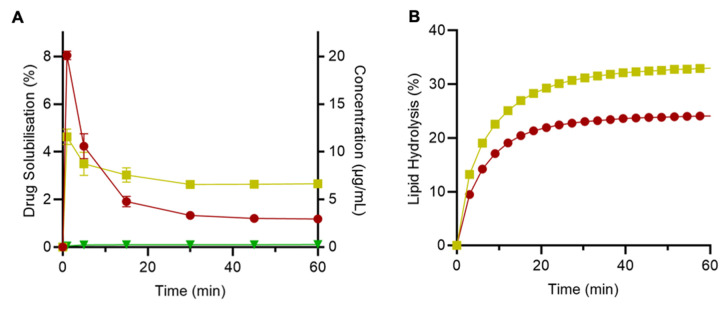
**(A)** *In vitro* solubilisation profiles under digesting conditions, and **(B)** lipolysis data for pure LUR [▼], LUR emulsion [●], and LUR-SLH [■]. Each value represents the mean ± SD, *n* = 3.

Similar to the previous dissolution study, LBFs were successful in significantly enhancing the solubilisation of LUR under digesting conditions, when compared to pure drug (Figure 6A). After the 60 min experimental period, LUR-SLH achieved the highest drug solubilisation of 2.7 ± 0.2% (6.6 μg/mL), compared to the LUR emulsion which reached 1.2 ± 0.0% (4.7 μg/mL) solubilisation. Only 0.1 ± 0.0% percent of the pure drug was solubilised in fasted state digesting conditions. The enhancement in LUR solubilisation from both LBFs is attributable to the digestion of the lipid component, which triggers the formation of colloidal species, composed primarily of mono- and di-glycerides and fatty acids. These colloidal lipid species, such as micelles, mixed micelles and vesicles, have demonstrated the ability to improve the dissolution of numerous poorly water-soluble drugs [7, 26], and the improved drug solubilisation during lipid digestion indicates that LUR is readily incorporated within these species.

*In vitro* lipase-mediated digestion of LUR LBFs, under simulated intestinal fasted state conditions, revealed that LUR-SLH enhanced lipolysis, compared to the LUR emulsion, by digesting 33.0 ± 0.3% of lipid hosted within the porous silica matrix (Figure 6B). In contrast, only 24.1 ± 0.2% of the LUR emulsion was digested over the course of 60 min lipolysis period. This is in accordance with previous studies that have highlighted the increased surface area of the porous silica matrix affords enhanced adsorption of lipase molecules in their active conformation, triggering an increase in catalytic activity [27]. Furthermore, owing to the negatively charged Aerosil® silica, the digestion of SLH has shown to trigger the release and repel the negatively charged free fatty acids from the nanostructured matrix into the aqueous phase [19]. The ability for SLH to promote lipid digestion and increase the concentration of fatty acid-rich solubilising vesicles within the aqueous phase are therefore considered the driving force for improved LUR solubilisation, when compared the LUR emulsion.

Drug precipitation is commonly observed with LBFs [28, 29]. Upon digestion of the exogenous lipid, the solubilisation capacity for the drug is enhanced and thus supersaturated drug concentrations may be generated [30]. The current solubilisation study was conducted in non-sink conditions and thus the lack of solubilising capacity of the aqueous phase resulted in rapid drug precipitation from LBFs, as evident within the initial 10 min of solubilisation (Figure 6A). However, precipitation during *in vitro* studies does not necessarily correlate with a reduction in bioavailability *in vivo,* as many studies suggests that this model over predicts the extent of drug precipitation due to the absence of an absorption compartment [31, 32].

#### Impact of RIS reformulation on lipolysis and drug solubilisation

**Figure 7.**
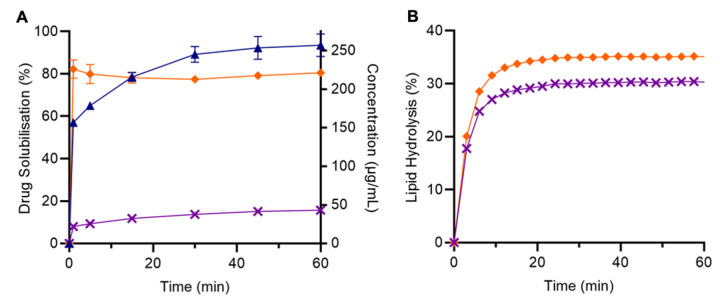
**(A)** *In vitro* solubilisation profiles under digesting conditions, and **(B)** lipolysis data for pure RIS [▲], RIS emulsion [◆], and RIS-SLH [✕]. Each value represents the mean ± SD, *n* = 3.

In contrast to LUR, pure RIS displayed the greatest level of drug solubilisation under digesting conditions, reaching a maximum of 93.4 ± 5.3% (254 μg/mL) after 60 min, followed by the RIS emulsion and RIS-SLH (Figure 7A). The drug solubilisation observed for pure RIS is indicative of the high solubility of the ionised drug in neutral conditions. A drug solubilisation level of 80.5 ± 0.8% (209 μg/mL), was observed for the RIS emulsion, likely owing to incomplete digestion of the lipid and thus, only partial drug partitioning to the aqueous phase. However, it is noteworthy that the performance of the RIS emulsion, relative to pure drug, was significantly greater during solubilisation studies under digestion conditions compared to non-digesting conditions, indicating the importance of lipase-provoked drug release. However, for RIS-SLH, a significant  2.2-fold reduction in solubilisation was evident, leading to a RIS solubilisation of only 43.2 ± 2.2% (109 μg/mL) after 60 min.

**Figure 8.**
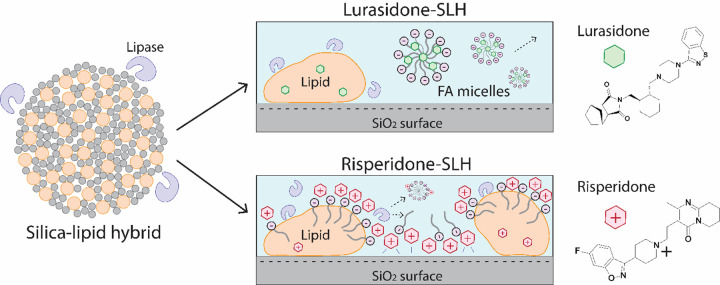
Schematic representation showing the difference in LUR and RIS solubilisation when encapsulated within SLH microparticles. At neutral pH, RIS is predominately ionised and carries a positive charge. As the silica matrix and fatty acids which are liberated upon lipase-mediated digestion of the lipid are negatively charged, complexation occurs between the ionised RIS and the silica surface, interfering with the availability for lipase-lipid interaction, and thus hindering lipid digestion and RIS solubilisation. Conversely, LUR is predominately unionised. Therefore, lipase can readily adsorb and digest the lipid, facilitating the production of negatively charged and highly solubilising LUR micelles which can be expelled into the aqueous phase due to repulsion from the negatively charged silica surface.

The reduced ability for RIS-SLH to promote solubilisation under digesting conditions can be attributed a reduction in lipolysis kinetics and the interaction between the ionised drug and the negatively charged silica surfaces and fatty acids produced during digestion. In contrary to the lipid digestion observed for LUR LBFs, the rate and extent of lipolysis was greatest for the RIS emulsion, with 34.9 ± 0.3% lipid being digested over the 60 min period, compared to only 30.2 ± 0.4% for RIS-SLH (Figure 7B). The difference in lipolysis behaviour between the two drugs, when encapsulated within SLH, is due to the relative degrees of ionisation, where RIS is mostly present in its ionised, positively charged form. Since the porous silica matrix and the fatty acids produced from lipolysis are both negatively charged, complexation occurs between ionised RIS and both the silica surface and lipid digestion products, as depicted in Figure 8. This interaction was confirmed by analysing the zeta potential of the various silica: drug: fatty acid complexes that are predicted to form during lipase-mediated digestion of both LUR- and RIS-SLH (Table 2). When porous silica particles were spiked with dissolved RIS, the zeta potential increased from -19.1 ± 2.7 mV to -12.3 ± 0.8 mV, highlighting the capacity for ionised RIS to adsorb to the negatively charged silica surface. However, once the silica: RIS complex was exposed to fatty acids, representative of those present during *in vitro* lipolysis (*i.e.* medium chain length fatty acids), the zeta potential again decreased to -15.0 ± 0.3 mV, indicating the propensity for negatively charged fatty acids to adsorb onto the RIS-coated silica surface through electrostatic interactions. In contrast, zeta potential variations when porous silica particles were exposed to LUR were statistically insignificant; thus, highlighting the ability for LUR and negatively charged fatty acids to be repelled from the bare silica surface (Table 2).

The adsorption of positively charged RIS molecules on the silica surface is therefore hypothesised to interfere with the ability for lipase to adsorb to the exposed silica surface in its active form, removing the potential to form a substrate-enzyme complex that enhances hydrolytic activity [33]. The digestion of lipid hosted within porous silica typically leads to the rapid expulsion of fatty acids from the silica pores, into the aqueous environment, due to an electrostatic repulsion interaction between the negatively charged silica surface and fatty acids. This process further enhances lipid digestion within SLH due to the absence of surface active, amphiphilic fatty acids at the lipid-in-water interface, which competitively inhibit lipase adsorption [27]. However, since ionised RIS can complex with fatty acids, their ability to be expelled into the aqueous phase is inhibited, and thus, they are likely to ‘spoil’ the lipid-in-water interface by retarding lipase adsorption (Figure 8), leading to reduced lipolysis kinetics.

Dening *et al.* recently characterised such drug-carrier and drug-fatty acid interactions when encapsulating ionisable drugs within solid-state LBFs composed of the smectite clay, montmorillonite, and medium chain triglycerides [34]. It was revealed that drug solubilisation under simulated intestinal digesting conditions for the model weak base, blonanserin, were reduced 3-fold compared to the pure drug, due to the electrostatic-driven interactions between ionised drug and montmorillonite and fatty acids. Inferior *in vitro* solubilisation performance correlated well with *in vivo* pharmacokinetics, whereby blonanserin bioavailability was reduced in montmorillonite-lipid hybrids, compared to the pure drug [35]. Thus, based on these findings, it may be expected that oral RIS bioavailability will also be reduced *in vivo*, when formulated with SLH. However, a greater dynamic flow is observed *in vivo,* and transcellular transport may be facilitated by uptake of the RIS-silica complex by intestinal cells; therefore, further analysis in animal models is necessary to determine whether performance *in vivo* will correlate with *in vitro* results for RIS-SLH.

### The Importance of Physicochemical Properties in Formulation Design

**Figure 9.**
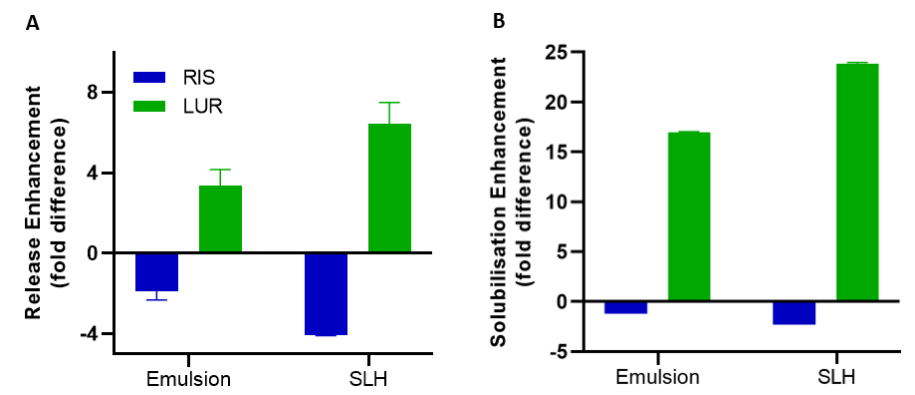
Formulation performance for RIS and LUR, emulsions and SLH, relative to pure drug, during **(A)** *in vitro* dissolution and **(B)** *in vitro* solubilisation under digestion conditions.

A summary of the impact of LBF type on RIS and LUR *in vitro* dissolution and solubilisation, relative to pure drug, is shown in Figure 9. A constant reduction in RIS performance is observed when RIS was formulated as a LBF, especially when fabricated as SLH. As described previously, this is a result from interactions between ionised RIS and negatively charged silica, thus hindering drug release. In contrast, such strong interactions were not present for LUR due to existing predominately in an unionised form.

The conflicting performance observed for RIS and LUR when formulated as a LBF demonstrates the importance of drug pKa and ionisation when developing a novel delivery system. A blanket statement has been used in literature surrounding the application of LBFs to overcome poor water-solubility and improve dissolution kinetics [4, 36, 37], however the performance of RIS diverges from this statement whereby a LBF hindered drug performance. Predictive criteria for determining suitable formulation approaches for poorly water-soluble compounds from current knoweldge is scarce, yet the present study reveals the need for a greater understanding of physicochemical parameters, such as pKa, in relation in performance when developing novel drug delivery systems.

## Conclusions

The poorly water-soluble antipsychotic drugs, RIS and LUR, have been successfully formulated and physicochemically characterised as lipid emulsions and SLH microparticles. The LBFs significantly improved the performance of LUR up to 23-fold during *in vitro* solubilisation studies in comparison to the unformulated drug, however a 2.2-fold reduction in solubilisation was observed for RIS due to its strong interaction with silica, demonstrating that the application of LBFs and the resulting performance may not be readily predicted. The results reveal that the pKa values of a compound must be considered during formulation development, and a general statement that LBFs may overcome the challenges of all poorly water-soluble drugs is not necessarily correct. Therefore, further investigation is required to develop predictive criteria to evaluate the performance of LBFs for poorly water-soluble compounds.

## Figures and Tables

**Table 1.** Formulation drug loading levels and encapsulation efficiencies (each value represents the mean ± SD, n = 3).

Formulation	Drug Loading (% w/w)	Encapsulation Efficiency (%)
RIS emulsion	3.12 ± 0.0	100 ± 0.0
RIS-SLH	1.64 ± 0.2	99.1 ± 1.3
LUR emulsion	1.4 ± 0.0	100 ± 0.0
LUR-SLH	0.75 ± 0.0	81.4 ± 0.0

**Table 2.** Zeta potential analysis of the various complexes formed between porous silica particles, drug molecules and fatty acids (each value represents the mean ± SD, n = 3).

Drug	Zeta potential (mV)		
	Porous silica particles (PSP)	PSP: drug complex	PSP: drug complex: FA complex
Lurasidone	-20.3 ± 3.3	-19.8 ± 1.6	-22.6 ± 4.2
Risperidone	-19.1 ± 2.7	-12.3 ± 0.8	-15.0 ± 0.3
